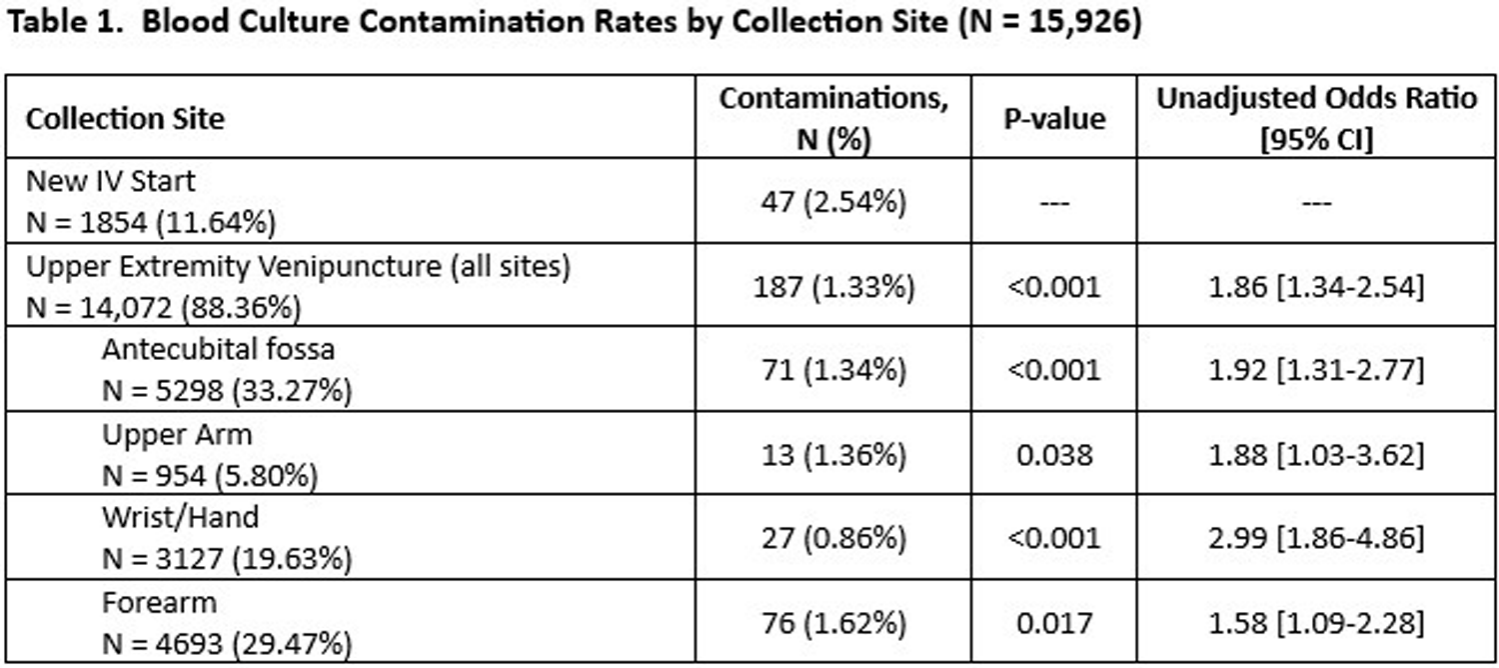# 367 Longitudinal trends in MDRO admission prevalence at Arizona hospitals among transfers from long-term care facilities: 2015-2023

**DOI:** 10.1017/ash.2026.10703

**Published:** 2026-06-23

**Authors:** Brandi Manning, Sydney Agnello, Shandra Day, Satomi Abe, Kelci Coe, Nora Colburn

**Affiliations:** 1 The Ohio State University; 2 Ohio State University; 3 The Ohio State University Wexner Medical Center; 4 Ohio State Wexner Medical Center

## Abstract

**Background:** Blood culture contamination leads to increased healthcare costs and patient harm. Routine practice at our institution oftentimes involves placement of a new peripheral intravenous catheter (IV) followed by immediate blood culture collection from the catheter. Published guidelines discourage routine blood culture collection from central venous catheters, but do not address collection from new IV starts. We hypothesized higher contamination rates in cultures collected from new IV starts. To evaluate, mandatory documentation of blood culture collection site was implemented and contamination rates were analyzed. **Methods:** A prospective observational study was conducted from September to December 2025 at a large quaternary medical center. Peripheral blood culture contamination rates were measured using the CLSI definition. Mandatory documentation of collection site was implemented September 2025 and included an option for “new IV start”. Contamination rates by collection site were compared using chi-squared tests. An estimated cost of $4,538 for each contamination event was based on pooled published cost analyses data. **Results:** During the study period, 15,926 peripheral blood culture sets were collected and 234 (1.47%) met contamination criteria. Table 1 shows the new IV start contamination rate was significantly higher than all other sites combined (p-value <0.001 with an odds ratio of 1.86 [1.34-2.54]). The difference remained when comparing new IV start to individual anatomic sites. Per year, an estimated 89 contaminations would be avoided if cultures are not obtained from new IV starts with an estimated savings of $403,882 and 267 antimicrobial days. **Conclusions:** At our institution, it is common practice to collect blood cultures immediately after inserting a peripheral IV. The contamination rate of new IV starts was nearly double the contamination rate of dedicated upper extremity venipuncture leading to increased costs and unnecessary antimicrobial use. The large sample size provides robust data that collecting blood cultures from a new IV start should be avoided and this compelling data should be used to update published guidelines.

**Figure:**